# Baseline periodontal status and modifiable risk factors are associated with tooth loss over a 10‐year period: Estimates of population attributable risk in a Japanese community

**DOI:** 10.1002/JPER.21-0191

**Published:** 2022-02-03

**Authors:** Michiko Furuta, Kenji Takeuchi, Toru Takeshita, Yukie Shibata, Shino Suma, Shinya Kageyama, Mikari Asakawa, Yoshihiro Shimazaki, Jun Hata, Toshiharu Ninomiya, Yoshihisa Yamashita

**Affiliations:** ^1^ Section of Preventive and Public Health Dentistry, Division of Oral Health, Growth and Development, Faculty of Dental Science Kyushu University Fukuoka Japan; ^2^ Department of Preventive Medicine Nagoya University Graduate School of Medicine Nagoya Japan; ^3^ OBT Research Center, Faculty of Dental Science Kyushu University Fukuoka Japan; ^4^ Department of Preventive Dentistry and Dental Public Health, School of Dentistry Aichi‐Gakuin University Nagoya Japan; ^5^ Department of Epidemiology and Public Health, Graduate School of Medical Sciences Kyushu University Fukuoka Japan

**Keywords:** health behavior, obesity, periodontitis, risk factors, tooth loss

## Abstract

**Background:**

This study aimed to examine whether modifiable risk factors can predict tooth loss over 10 years and estimate population attributable risk (PAR) for a combination of modifiable factors.

**Methods:**

This longitudinal study included 1466 participants who underwent dental examinations in 2007 and 2017 and were aged 40 to 79 years at baseline. Periodontal conditions were assessed using the 2018 periodontal classification. Incident tooth loss was defined as ≥4 teeth lost over a 10‐year period. We calculated the partial PAR (pPAR%) for tooth loss to estimate the combined effect of modifiable risk factors.

**Results:**

Incidence of tooth loss was 17.5%. Directed acyclic graphs were used to identify risk factors for tooth loss. A logistic regression model showed that baseline periodontitis, dental caries experience, no regular dental visit, periodontal treatment, smoking, and obesity were associated with tooth loss after adjusting for covariates; pPAR% was 55.5% (95% confidence interval: 31.1% to 73.0%) in periodontitis Stage III to IV and 87.6% (50.4% to 97.4%) in the combination of all factors, respectively. The sex‐stratified analysis showed that smoking and no regular dental visit in men and obesity in women were identified as potential risk factors for tooth loss.

**Conclusions:**

Modifiable factors accounted for most cases of incident tooth loss. Risk factors for tooth loss might differ by sex, suggesting that the appropriate approach for preventing tooth loss base on sex.

## 1 INTRODUCTION

The number of teeth is an effective marker of oral function as well as overall health. Poor dentition attenuates chewing ability^1^ and causes self‐limiting food selection, resulting in poor nutritional status.^2^ Additionally, research has shown that having fewer teeth is associated with cognitive^3^ and physical function decline.^4^ A large cohort study of community‐dwelling people aged 85 years and older revealed that those with 20 or more teeth had a longer and healthier life expectancy than edentulous people.^5^

Tooth loss is the endpoint of oral diseases, such as dental caries and periodontitis, and reflects utilization of dental services during a lifetime. The incidence and prevalence of total tooth loss has significantly declined at the global, regional, and country levels.^6^ However, tooth loss and oral diseases are still a major public health concern worldwide.^7^ Most individuals begin to lose their teeth in their middle age. A national survey in Japan during 2016 reported that the percentage of individuals who had at least one missing tooth was 35.5% for 40‐ to 49‐year‐olds, 67.6% for 50‐ to 59‐year‐olds, 84.3% for 60‐ to 69‐year‐olds, and 89.0% for 70‐ to 79‐year‐olds.^8^ These data indicate that almost all middle‐aged and older Japanese individuals experience tooth loss. Therefore, increased attention should be given to the risk factors for tooth loss within these age groups.

Although identification of risk factors can be helpful to find preventive strategies, policy making also requires information on the potential impact of the risk factors in populations. This impact can be quantified as the population attributable risk (PAR), which considers not only the strength of the association but also the prevalence of exposure.^9^ The partial PAR (pPAR) can estimate the PAR of modifiable factors and combinations of factors.^10^ Tooth loss has a multifactorial etiology and is influenced by sociodemographic and lifestyle factors, oral health behavior, and systemic health.^11^, ^12^, ^13^ To date, no study has demonstrated the PAR of a combination of risk factors for tooth loss. As these factors tend to cluster within populations, understanding the combined effects of these factors could be informative for policy making and resource allocation focused on primary prevention.

The objectives of this study were to investigate the association of periodontitis, dental caries, and risk factors with tooth loss over a 10‐year period and to quantify the combined risk of these factors by assessing the pPAR in community‐dwelling people.

## 2 MATERIALS AND METHODS

### 2.1 Study population

**FIGURE 1:**
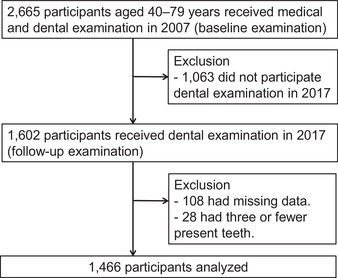
Flow diagram of the study participants

This study was conducted as a part of the Hisayama Study, a population‐based prospective study of cardiovascular disease in the town of Hisayama, a suburb of the Fukuoka metropolitan area in southern Japan. The demographic characteristics of the study cohort, such as age and occupation, were similar to the general Japanese population.^14^ Dental surveys were conducted on Hisayama residents aged 40 to 79 years in 2007 and 2017. The study population included 2,665 participants, who underwent both medical and dental examinations in 2007 (70.0% of all residents in this age group).^15^ Similarly, the number of participants was 2,285 (54.1%) in 2017. Among the participants in 2007, 1602 participants underwent dental examinations in 2017 (follow‐up rate = 60.1%). Of them, 108 participants with missing data and 28 participants with three or fewer present teeth in 2007 were excluded. The outcome in this study was tooth loss defined by four or more teeth lost (highest quintile of the number of teeth lost) over a 10‐year period. Third molars were excluded from these analyses. Tooth loss was evaluated by the difference between the number of present teeth at baseline (in the 2007 survey) minus the number of present teeth in the 2017. In participants with three or fewer teeth at baseline, the maximum number of teeth lost was three, which never resulted in four or more teeth lost (incident tooth loss). Therefore, these participants were excluded. Finally, 1,466 participants (630 men and 836 women) were examined for tooth loss over 10 years (Figure 1). The study protocol was approved by the Kyushu University Institutional Review Board for Clinical Research (Approval No. 28‐31). Informed written consent was obtained from all participants.

### 2.2 Assessment of oral health condition

Evaluation of oral health condition included the number of present teeth, periodontal condition, and dental caries experience. The total number of decayed and filled teeth (DFT) was used to measure dental caries. Examination of the periodontium followed the National Health and Nutrition Examination Survey (NHANES) III method which included assessment of probing pocket depth (PPD) and clinical attachment level (CAL) of all the teeth (except the third molars), at two sites (mesiobuccal and mid‐buccal)^16^ by using a periodontal probe.^1^ The percentage of teeth that bled upon probing (%BOP) was also assessed. The mean values for PPD and CAL were calculated as the sum of the maximum PPD or CAL per tooth, divided by the number of present teeth in each individual. A total of nine trained dentists (TT, YS, and YS and Sumio Akifusa, Miki Kawada, Noriaki Kamio, Masaki Yasui, Nao Fukui, and Mikiko Tomioka, Faculty of Dental Science, Kyushu University, Fukuoka, Japan) assessed the periodontal condition. To ensure the reliability of periodontal measurements, the calibration was performed by conducting examinations on volunteers prior to the start of the study. The inter‐examiner agreement for PPD measurements within ±1.0 mm between eight research examiners and the other examiner (YS) as the gold‐standard examiner was very high (kappa > 0.8).^15^ The intra‐class correlation coefficients, used as a measure of inter‐examiner reproducibility, were 0.873 and 0.862 for PD and CAL, respectively.

Periodontitis was diagnosed according to the classification proposed at the 2018 World Workshop on the classification of periodontal and peri‐implant diseases and conditions.^17^, ^18^ Periodontitis was defined as interdental clinical attachment loss at ≥2 non‐adjacent teeth or buccal or oral clinical attachment loss ≥3 mm with pocketing ≥3 mm on ≥2 teeth.^17^ After excluding periodontal health (defined by < 10% BOP with PPD ≤3 mm) and gingivitis (defined by ≥ 10% BOP with PPD ≤3 mm),^19^, ^20^ staging of periodontitis were defined. Regarding staging, clinical attachment loss of 1‐2 mm was defined as Stage I, 3‐4 mm was defined as Stage II, and ≥5 mm was defined as Stage III.^18^ By assessing maximum PPD (Stage I, PPD ≤4 mm; Stage II, PPD ≤5 mm; Stage III, PPD ≥6 mm), stage‐shifting was possible only when the stage increased.^18^ For example, Stage II would shift to Stage III if the maximum PPD was 6 mm or greater. Reasons for missing teeth were not examined in this study. Therefore, stage shifting complexity factors including the number of present teeth were considered, to discriminate between Stages III and IV. Less than 20 present teeth were classified as Stage IV.^18^

### 2.3 Clinical and biochemical assessments

In the 2007 survey, clinical and biochemical assessments included measurements of plasma glucose level, systolic and diastolic blood pressure, serum triglycerides, serum high‐density lipoprotein (HDL) cholesterol concentrations, and body mass index (BMI).^21^ Blood samples were collected after overnight fasting. All participants in this study, except for those with severe diabetes or undergoing insulin treatment, received a 75‐g oral glucose tolerance test. Plasma glucose concentrations were determined using the hexokinase method. Diabetes was defined as either undergoing treatment for diabetes with medication and/or insulin injections, or a fasting plasma glucose ≥126 mg/dL, 2 hours post‐prandial and random plasma glucose ≥200 mg/dL, or both.^22^ Hypertension was defined as systolic blood pressure ≥130 mmHg, diastolic blood pressure ≥80 mmHg, or currently undergoing antihypertensive treatment in the US guidelines,^23^ and systolic blood pressure ≥140 mmHg, diastolic blood pressure ≥90 mmHg, or ongoing antihypertensive treatment in European guidelines.^24^ Obesity was defined as BMI ≥ 25.0 kg/m^2^, which was the optimal cut‐off for obesity in Asian individuals.^25^

### 2.4 Questionnaire

Information about participants’ smoking habits, occupational status, tooth brushing frequency, regular dental visits, periodontal treatment was obtained using a self‐administered questionnaire. Smoking status was divided into current smoker, former smoker and never smoked. Occupational status was classified into three categories: clerical support workers, homemaker, unemployed or retired, and other jobs. The frequency of tooth brushing was categorized as once per day or less, and twice per day or more. Participants were categorized as those who did or did not regularly visit the dentist for oral care at least once a year. Periodontal treatment was dichotomized as periodontal treatment within 1 year/over 1 year ago versus no/do not know.

### 2.5 Statistical analysis

We used the chi‐square (χ^2^) test for categorical variables, the *t‐*test for normally distributed variables, and the Mann‐Whitney *U* test for non‐normally distributed variables with significance defined as *P* < 0.05, two‐tailed testing.

The directed acyclic graphs (DAG) (SAS CAUSALGRAPH; SAS Institute, Cary, NC) was used to identify the minimum set of variables that need to be included in the multivariable models. Supplemental Figure 1 in the online *Journal of Periodontology* illustrates the DAG representing baseline risk factors as the exposure variables, periodontitis and number of DFT as mediator, and tooth loss as outcomes. Modifiable factors were regarded as risk factors in this study. Risk factors included regular dental visit, periodontal treatment, tooth brushing, smoking, obesity, and hypertension as categorical variables, triglyceride and HDL cholesterol as continuous variables. In the current medical care system, it remains difficult to completely improve diabetes outcomes.^26^ It was considered that diabetes was not modifiable factor. In this study, diabetes was used as a covariate. The covariates were age, sex, occupational status, diabetes, and number of present teeth. The multivariable model included the variables identified by the DAG.

Multivariate logistic regression was used to assess baseline risk factors that were considered to be related to tooth loss. Odds ratios (ORs) and 95% confidence intervals (CIs) were calculated using the logistic regression model. In this model, quintiles of the number of teeth lost during the 10‐year period were divided into two groups: highest quintile (≥ 4 teeth lost) vs. the four lower quintiles (≤ 3 teeth lost) (Supplemental Figure 2). We evaluated three different models as follows: (1) Model 1 included baseline age, sex, and one risk factor for tooth loss; (2) Model 2 included baseline age, sex, occupational status, diabetes, number of present teeth, and all risk factors except periodontal treatment; (3) Model 3 included variables in Model 2 plus periodontal treatment at baseline. Additionally, a separate analysis by sex was performed. Crude ORs were compared between men and women using the Breslow‐Day test. To evaluate the consistency of the results obtained using highest quintiles of the number of teeth lost as an outcome, the association between the number of teeth lost as a continuous count variable and risk factors was investigated using zero‐inflated Poisson regression models.

In the risk factors that showed a significant association in the logistic regression model (Model 3), including all factors identified by DAG, the pPAR and 95% CI for tooth loss related factors were calculated using the methods and %PAR macro described by Spiegelman et al.^10^ When we calculated the pPAR, the risk factor with no association in the logistic regression model was included in all models because this factor may be correlated with other risk factors. The pPAR estimates the percentage of cases that can be prevented if modifiable risk factors are eliminated while keeping other non‐modifiable risk factors unchanged. In our analysis, the fixed factors included age, sex, occupational status, and diabetes. Additionally, we used the pPAR to estimate PAR for different combinations of risk factors. Tooth loss is considered to have multiple risk factors, and pPAR is useful to understand the combined effects of risk factors on tooth loss.

To assess whether the models are adequate for predicting tooth loss, sensitivity, specificity, positive predictive value, negative predictive value, and area under the curve (AUC) were calculated using a logistic regression model.

We used logistic mixed models to evaluate the association of changes in risk factors with tooth loss. The 2017 data on periodontitis, number of DFT, no regular dental visit, and tooth brushing were available, and the changes in these factors except number of DFT were treated as time‐varying variables. The reason for excluding number of DFT was that tooth extraction due to dental caries results in a decreasing number of DFT, meaning that this change is caused by tooth loss. The model was then adjusted for baseline age, sex, and number of present teeth. The statistical software^2^ was used to perform all statistical analyses.

## 3 RESULTS

**FIGURE 2:**
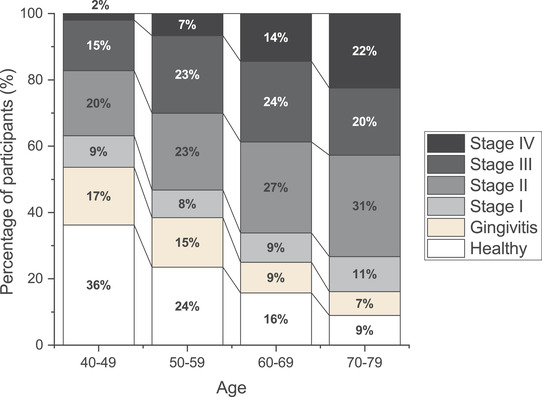
Distribution of periodontitis stage in the 2018 classification according to age group in study population (*n* = 2,569) Of 2665 participants who received dental examination in 2007, periodontal examination was conducted in 2569 participants excluding edentulous

Periodontitis stage in the study population according to age groups is shown in Figure 2. The percentage of individuals with Stage III or IV was 17.2% in age groups of 40 to 49 years, 30.1% in 50 to 59 years, 38.8% in 60 to 69 years, 42.7% in 70 to 79 years. Differences in oral and systemic health conditions in 2007 were compared between the individuals included and excluded from the analyses (Table 1). There were significant differences between the two groups with respect to age, number of present teeth and DFT, periodontal condition, oral health behavior, current smoking, diabetes, hypertension, triglycerides, and occupational status (Table 1). Moreover, as compared to participants who were not analyzed because of missing data and no incidence of tooth loss (three or fewer present teeth at baseline), analyzed participants were younger and had more present teeth and DFT (see Supplementary Table 1 in online *Journal of Periodontology*).

**TABLE 1:** Descriptive statistics in 2007 comparing analyzed participants with participants who were not analyzed

Baseline variable	Analyzed (*n *= 1,466)	Not analyzed (*n *= 1,199)*	*P* value
Age, years	58.3 ± 9.5	63.4 ± 10.7	< 0.001
Women, %	57.0	54.1	0.134
Number of present teeth excluding third molar	24.3 ± 4.5	18.8 ± 8.8	< 0.001
Number of DFT	14.5 ± 5.5	12.4 ± 6.8	< 0.001
Mean PPD^†^, mm	2.24 ± 0.71	2.52 ± 0.91	< 0.001
Mean CAL^†^, mm	2.54 ± 0.89	3.02 ± 1.20	< 0.001
Periodontitis^†^			< 0.001
No, gingivitis	36.8	25.9	
Stage I, II	34.3	35.1	
Stage III	22.2	20.7	
Stage IV	6.7	18.3	
Toothbrushing ≤ 1 time^‡^, %	29.1	37.7	< 0.001
No regular dental visit^§^, %	70.7	76.2	0.001
Periodontal treatment^‖^, %	29.3	31.5	0.204
Current smoking, %	18.8	23.4	0.004
Diabetes, %	13.5	20.5	< 0.001
Obesity (BMI ≥ 25.0), %	25.6	28.9	0.083
Hypertension definition (American), %	58.2	67.7	< 0.001
Hypertension definition (European), %	40.7	52.3	< 0.001
Serum triglycerides, mg/dL	124.4 ± 100.9 100.0 (72.0, 141.0)	131.4 ± 110.8 101.0 (75.0, 153.0)	0.023
Serum HDL cholesterol, mg/dL	67.4 ± 17.5	66.8 ± 18.5	0.308
Occupational status			< 0.001
Clerical support workers, %	27.9	25.4	
Other jobs, %	24.0	18.0	
Homemaker, unemployed or retired, %	48.1	56.6	

All variables except serum triglycerides are given as the mean ± standard deviation or as a percentage. Serum triglycerides are given as mean ± standard deviation and median (first quartile, third quartile).

Chi‐square test was performed for categorical variables, and t‐test was performed for continuous variable except serum triglycerides. Mann‐Whitney *U* test was performed for serum triglycerides.

* Participants who did not receive dental examination in 2017 (n = 1,063), had missing data (n = 108), and had three or fewer present teeth (n = 28).

^†^ Excluding edentulous (n = 96 in participants who were not analyzed).

^‡^ Excluding individuals with missing value (n = 17 in participants who were not analyzed).

^§^ Excluding individuals with missing value (n = 50 in participants who were not analyzed).

^‖^ Excluding individuals with missing value (n = 10 in participants who were not analyzed).

DFT, decayed and filled teeth; PPD, probing pocket depth; CAL, clinical attachment level; BMI, body mass index; HDL, high‐density lipoprotein.

Supplementary Figure 2 shows the distribution of the number of teeth lost during a 10‐year period from 2007 to 2017. The incidence of participants with four or more teeth lost in the decade was 17.5%.

The factors based on DAG for minimum adjustment were as follows:　periodontitis stage, number of DFT, tooth brushing, regular dental visit, periodontal treatment, smoking, obesity, diabetes, age, sex, occupational status, and number of present teeth. When we analyzed using definition of hypertension in US or Europe guidelines, hypertension was not included in the minimum adjustment set of covariates. Periodontitis stage, number of DFT, tooth brushing, regular dental visit, periodontal treatment, smoking, and obesity were used as the modifiable factor.

The logistic regression model found that the association of no regular dental visit with tooth loss was not significant in Model 2, but it was significant in Model 3 which added periodontal treatment. The highest quintile of number of teeth lost in the decade was positively associated with no regular dental visit (OR 1.63, 95% CI 1.13–2.35), periodontal treatment (OR 2.06, 95% CI 1.47–2.87), current smoking (OR 1.69, 95% CI 1.10–2.60) and obesity (OR 1.66, 95% CI 1.17–2.35) at baseline (Model 3 in Table 2). This model included age, sex, occupational status, diabetes, number of present teeth, and all the modifiable factors. When the validation parameters were evaluated based on Model 3, sensitivity and specificity were 84.4% and 66.3%, respectively (Supplementary Table 2). The positive and negative predictive values were 34.8% and 95.2%, respectively, and the AUC was estimated to be 0.841 (Supplementary Table 2).

**TABLE 2:** Odds ratio of tooth loss during 10‐year period according to modifiable risk factors

				Adjusted OR (95%CI)		
Baseline variable	n	Incidences (%)	Crude OR (95%CI)	Model 1	Model 2	Model 3
**Periodontitis**						
No, gingivitis	540	3.7	1	1	1	1
Stage I, II	503	15.9	4.92 (2.96‐8.16)	3.95 (2.36‐6.60)	3.15 (1.85‐5.34)	3.09 (1.81‐5.26)
Stage III	325	28.6	10.42 (6.28‐17.31)	8.31 (4.95‐13.95)	7.97 (4.68‐13.60)	7.32 (4.27‐12.53)
Stage IV	98	65.3	48.94 (26.58‐90.10)	31.53 (16.80‐59.19)	9.65 (4.73‐19.68)	8.61 (4.20‐17.68)
**Number of DFT**	1466		0.91 (0.90‐0.91)	0.99 (0.97‐1.02)	1.06 (1.02‐1.09)	1.06 (1.02‐1.09)
**Toothbrushing**						
≥ 2 times	1040	17.2	1	1	1	1
≤ 1 time	426	18.3	1.08 (0.80‐1.45)	0.89 (0.65‐1.22)	0.79 (0.55‐1.13)	0.81 (0.57‐1.16)
**Regular dental visits**						
Yes	430	17.0	1	1	1	1
No	1036	17.8	1.06 (0.78‐1.42)	1.29 (0.95‐1.76)	1.34 (0.94‐1.91)	1.63 (1.13‐2.35)
**Periodontal treatment**						
No/unknown	1037	12.7	1	1		1
Yes	429	29.1	2.82 (2.14‐3.72)	2.31 (1.73‐3.08)		2.06 (1.47‐2.87)
**Smoking**						
Past/never	1191	16.2	1	1	1	1
Current	275	23.3	1.57 (1.14‐2.16)	2.19 (1.51‐3.18)	1.63 (1.07‐2.49)	1.69 (1.10‐2.60)
**Obesity** (BMI ≥ 25.0)						
No	1087	15.7	1	1	1	1
Yes	379	22.7	1.57 (1.18‐2.10)	1.58 (1.17‐2.14)	1.62 (1.15‐2.29)	1.66 (1.17‐2.35)

Logistic regression models; tooth loss (highest quintile of number of teeth lost during a 10‐year period [≥ 4 teeth lost]) was the dependent variable and risk factors were the independent variable.

Model 1 included age, sex, and one risk factor.

Model 2 included age, sex, occupational status, diabetes, number of present teeth and modifiable factors such as toothbrushing frequency, regular dental visits, smoking, obesity, periodontitis, and number of DFT.

Model 3 included variables in model 2 plus periodontal treatment.

DFT, decayed and filled teeth; BMI, body mass index; OR, odds ratio; CI, confidence interval.

Table 3 shows pPAR for tooth loss for various modifiable risk factors. The pPAR analysis included periodontitis stage, number of DFT, no regular dental visit, periodontal treatment, current smoking, and obesity at baseline. Among the individual contributions of the various modifiable risk factors, periodontitis stage contributed to tooth loss (pPAR 55.5%, 95% CI 31.1–73.0%), followed by periodontal treatment (pPAR 32.4%, 95% CI 18.8–44.8%). In combination, three risk factors (no regular dental visit + smoking + obesity) contributed 36.9% (95% CI 7.2–60.7%) in the pPAR of tooth loss, whereas all factors contributed 87.6% (95% CI 50.4–97.4%).

**TABLE 3:** Partial population attributable risk for individual risk factors and combinations of risk factors for tooth loss

Risk factors	pPAR%*	(95% CI)
*Individual modifiable risk factors* ^†^		
Periodontitis Stage III, IV	55.5	(31.1, 73.0)
Number of DFT	19.5	(1.5, 36.2)
No regular dental visit	18.5	(‐0.01, 0.37)
Periodontal treatment	32.4	(18.8, 44.8)
Current smoking	13.4	(6.8, 19.9)
Obesity (BMI ≥ 25.0)	12.7	(2.2, 22.9)
*Combination of modifiable risk factors* ^‡^		
Risk factors combination 1 = no regular dental visit + current smoking + obesity	36.9	(7.2, 60.7)
Risk factors combination 2 = risk factor combination 1+ periodontal treatment	65.9	(37.6, 83.0)
Risk factors combination 3 = risk factor combination 2 + number of DFT	74.5	(42.8, 89.9)
Risk factors combination 4 = risk factor combination 3 + periodontitis	87.6	(50.4, 97.4)

* Adjusted for toothbrushing frequency, age, sex, occupational status, diabetes, and number of present teeth.

^†^ Model included each modifiable risk factor and covariates.

^‡^ Model included multiple risk factors and covariates.

DFT, decayed and filled teeth; BMI, body mass index; pPAR, partial population attributable risk; CI, confidence interval.

The percentage of participants with ≥ 4 teeth lost during a 10‐year period was 21.1% in men and 14.8% in women. We found the sex difference in number of DFT, periodontal condition, oral health behavior, diabetes, and obesity (Supplementary Table 3), and therefore we conducted stratified analysis by sex. Current smoking and no regular dental visit remained a significant factor for tooth loss in men, whereas in women, obesity was significant factor (Table 4). In the pPAR of tooth loss, current smoking in men contributed 26.2% (95% CI 12.5–39.0%), and obesity in women contributed 22.2% (95% CI 5.5–37.7%).

**TABLE 4:** Odds ratio of tooth loss during 10‐year period according to risk factors in men and women

	Men				Women			
Baseline variable	Incidence (%)	Crude OR (95% CI)	Adjusted OR (95% CI)	pPAR% (95% CI)	Incidence (%)	Crude OR (95% CI)	Adjusted OR (95% CI)	pPAR% (95% CI)
**Periodontitis**								
No, gingivitis, Stage I, II	9.9	1	1		9.4	1	1	
Stage III	29.8	3.86 (2.44‐6.11)	3.20 (1.94‐5.27)		27.0	3.56 (2.25‐5.64)	3.60 (2.16‐6.02)	
Stage IV	67.2	18.69 (9.83‐35.54)	2.82 (1.14‐6.96)	65.0 (18.8, 87.7)	62.5	16.05 (8.04‐32.04)	4.39 (1.95‐9.84)	45.8 (9.8, 71.2)
**Number of DFT**		0.92 (0.90‐0.93)	1.05 (1.01‐1.09)	16.5 (‐0.04, 35.4)		0.90 (0.89‐0.91)	1.08 (1.02‐1.14)	31.4 (1.6, 56.0)
**Toothbrushing**								
≥ 2 times	20.8	1	1		15.3	1	1	
≤ 1 time	21.5	1.04 (0.71‐1.53)	0.95 (0.60‐1.50)		12.8	0.81 (0.49‐1.36)	0.60 (0.33‐1.09)	
**Regular dental visit**								
Yes	18.3	1	1		16.1	1	1	
No	22.0	1.28 (0.82‐1.98)	1.80 (1.04‐3.10)	27.0 (‐0.01, 0.51)	14.3	0.87 (0.58‐1.31)	1.56 (0.94‐2.57)	
**Periodontal treatment**								
No	15.2	1	1		10.9	1	1	
Yes	34.0	2.87 (1.93‐4.25)	2.23 (1.39‐3.60)	34.5 (13.1, 52.8)	25.0	2.72 (1.84‐4.02)	2.05 (1.28‐3.28)	30.9 (10.2, 48.4)
**Smoking**								
Past/never	18.3	1	1		15.1	1	1	
Current	26.5	1.61* (1.09‐2.38)	1.89 (1.15‐3.12)	26.2 (12.5, 39.0)	11.7	0.74 (0.33‐1.68)	1.22 (0.47‐3.13)	
**Obesity** (BMI ≥ 25.0)								
No	20.8	1	1		12.3	1	1	
Yes	21.8	1.06* (0.70‐1.61)	1.23 (0.75‐2.02)		23.6	2.21 (1.47‐3.32)	2.24 (1.37‐3.66)	22.2 (5.5, 37.7)
								
*Combination of modifiable risk factors*								
No regular dental visit + periodontal treatment + current smoking + number of DFT + periodontitis				80.2 (40.8, 94.4)				
Periodontal treatment + obesity + number of DFT + periodontitis								82.2 (22.7, 97.0)

Logistic regression models; tooth loss (highest quintile of number of teeth lost during a 10‐year period [≥ 4 teeth lost]) is the dependent variable and risk factors are the independent variables.

* Significant differences compared to women (*P* < 0.05).

The model included age, occupational status, number of present teeth, toothbrushing frequency, periodontal treatment, smoking, diabetes, obesity, periodontitis, and number of DFT.

DFT, decayed and filled teeth; BMI, body mass index; OR, odds ratio; CI, confidence interval.

When tooth loss was used as a continuous variable, the number of teeth lost according to the periodontitis stage is shown in Supplementary Table 4. The associations between risk factors and the number of teeth lost were similar to the results of tooth loss as dichotomous variable (Supplementary Table 5).

We evaluated changes in oral condition and health behaviors (Supplementary Table 6) and the association between changes in these factors and tooth loss (Supplementary Table 7). The logistic mixed model showed that an increase in periodontitis Stage IV were positively associated with tooth loss (Supplementary Table 7).

## 4 DISCUSSION

This present study reports the relative contributions of various modifiable risk factors, singly and in combination, to tooth loss incidence in Japanese community residents. We estimated that 87.6% of tooth loss could be prevented by modifying identifiable risk factors.

In most previous studies regarding tooth loss, health behavior factors and systemic heath have been considered individually,^11^, ^12^, ^13^ although these factors are typically correlated. When we simultaneously examined a set of these factors, no regular dental visit, periodontal treatment, smoking, and obesity accounted for 65.9% of incident tooth loss (Table 3). The results of pPAR may help to develop public health interventions and prevention‐oriented clinical practices. The pPAR of the combination of risk factors may also indicate population subgroups in particular need for intervention for promoting oral health. Based on the results of pPAR, the accumulation of risk factors increases the risk of tooth loss. Interventions for individuals with a high number of risk factors, such as periodontal treatment, no regular dental visit, smoking, or obesity may be effective in preventing further tooth loss.

A clinical‐based study has confirmed the possibility that the periodontitis stage can predict periodontitis‐related tooth loss, as well as total tooth loss.^27^ Total tooth loss was evaluated in this study; pPAR for tooth loss was 55.5% in the periodontitis stage and 19.5% in the number of DFT. A nationwide survey among dental clinics in Japan on reasons for tooth extraction has shown that the same percentage of teeth was extracted due to periodontitis (34.3%) and caries (34.4%) at 40 to 59 years of age, whereas the percentage of teeth extracted due to periodontitis (48.8%) was higher than that extracted due to caries (22.7%) in those aged 60 to 79 years.^28^ A higher pPAR in the periodontitis stage than in dental caries may be explained by the higher number of teeth lost due to periodontitis rather than caries at advanced ages. Although tooth loss due to periodontitis may be directly relevant to the new classification of periodontitis,^18^ staging could explain more than half of all recorded cases of incident tooth loss over 10 years.

Stratified analysis by sex indicates that the risk factor for tooth loss is different between men and women. Current smoking was potential risk factors for tooth loss in men. Obesity was risk factors for tooth loss in women. It appears that health behavior in men and systemic health in women were related to tooth loss. In particular, the prevalence of obesity was lower in women than men, and however, the association between obesity and tooth loss was observed in women. Women with obesity had higher levels of C‐reactive protein than men, suggesting that the systemic inflammation level was higher in women than in men with obesity.^29^ Periodontitis, which is a leading cause of tooth loss, is associated with systemic inflammation. The association of higher inflammation with obesity could explain the stronger association between systemic health and tooth loss in women than in men. This difference requires consideration of sex‐specific approaches for the prevention of tooth loss.

The percentage of participants with ≥ 4 teeth lost during a 10‐year period was found to be significantly higher in men than in women in this study (21.1% in men and 14.8% in women). There is a strong likelihood that a motivated recommendation to quit smoking and regular dental visit in men, can lead to reduced tooth loss in the population. In view of the approaches made by dentists’ for tooth loss prevention, recommending the patients to quit smoking and regular dental visit would be an easier approach to contribute to preventing tooth loss than controlling obesity. Furthermore, in a dental setting, measurement of weight and height to monitor obesity might be logistically difficult and inconvenient for dentists and patients.

The mean number of teeth lost over 10 years was 2.70 in periodontitis Stage III and 5.52 in Stage IV in this study (Supplementary Table 4). A recent systematic review reported that periodontitis patients who underwent active periodontal therapy and long‐term periodontal maintenance care lost one tooth over 10 years.^30^ Our participants might not have received periodontal maintenance care for a long period. For patients with Stage III and IV periodontitis, long‐term periodontal maintenance care is considered a highly effective approach to prevent further tooth loss.

This study had several limitations. First, the reasons for tooth extraction (e.g., dental caries or periodontitis) were not investigated because of the limited examination time. Additionally, the use of dental floss and interdental brush was not investigated in the 2007 survey, therefore, we could not evaluate the effect of dental floss and interdental brush use on tooth loss. Second, some factors such as socioeconomic status may affect the dentist's decision to extract a tooth. This may cause a bias in the observed association between risk factors and tooth loss. However, the Japanese public health insurance system covers almost all dental therapies, therefore, socioeconomic status may have little effect in Japan. Third, we used a partial‐mouth assessment for periodontal condition, which did not include an examination of lingual or palatal sites. Our results potentially underestimate periodontal conditions. However, full‐mouth partial diagnostic records in the 2018 classification of periodontitis were found to be effective for estimating prevalence and severity.^31^ Fourthly, we did not assess the grade of periodontitis according to the 2018 classification system, because we did not obtain data on radiographic bone loss or CAL 5 years prior to baseline. Finally, the follow‐up rate in this study was 60% becaude the older participants were less likely to receive dental examination in 2017. Oral and systemic health was worse in participants who were not analyzed than in those who were (Table 1). This limitation should be taken into consideration when applying these findings to other populations.

## 5 CONCLUSION

This study demonstrated that the majority of tooth loss was attributable to potentially modifiable risk factors. The risk factors for tooth loss were sex‐dependent. Reduction of smoking prevalence and increasing regular dental visits in men and reduction of obesity prevalence in women would appear to offer the benefit of preventing tooth loss in the population. The present study data may have important public health implications for preventing tooth loss.

## CONFLICTS OF INTEREST

The authors declare that they have no conflicts of interest.

## AUTHOR CONTRIBUTIONS

Michiko Furuta, Kenji Takeuchi, Toshiharu Ninomiya, and Yoshihisa Yamashita conceived and designed the study. Michiko Furuta and Kenji Takeuchi conducted statistical analysis. Michiko Furuta, Kenji Takeuchi, Toru Takeshita, Jun Hata, Toshiharu Ninomiya, and Yoshihisa Yamashita interpreted the data and drafted the article. All authors acquired the data and critically reviewed and approved the final manuscript.

## Supporting information



Supplementary material

Supplementary material

Supplementary material

Supplementary material

Supplementary material

Supplementary material

Supplementary material

Supplementary material

Supplementary material

## Footnotes

1 PCP11, Hu‐Friedy, Chicago, IL.

2 The Statistical Analysis System software package version 9.4, SAS Institute, Cary, NC.
