# Supplementary material for: Baseline periodontal status and modifiable risk factors are associated with tooth loss over a 10‐year period: Estimates of population attributable risk in a Japanese community
Source: J Periodontol. 2022 Feb 3;93(4):526–36. doi: 10.1002/JPER.21-0191 (PMC9305417; doi:10.1002/JPER.21-0191)
Supplement: Supplementary file 1 — Supplementary material [file JPER-93-526-s005.docx]

| Supplementary Table 1. Descriptive statistics in 2007 comparing analyzed participants with participants who were not analyzed because of missing data and no incidence of tooth loss (three of fewer present teeth at baseline). | | | |
| --- | --- | --- | --- |
| Baseline variable | Participants who was analyzed  (n = 1,466) | Participants who were not analyzed because of missing data and no incidence of tooth loss  (n = 136) | p value |
| Age, years | 58.3 ± 9.5 | 63.2 ± 9.4 | < 0.001 |
| Women, % | 57.0 | 57.4 | 0.941 |
| Number of present teeth excluding third molar | 24.3 ± 4.5 | 18.1 ± 10.1 | < 0.001 |
| Number of DFT | 14.5 ± 5.5 | 11.3 ± 7.5 | < 0.001 |
| Mean PPD^*^, mm | 2.24 ± 0.71 | 2.20 ± 0.86 | 0.716 |
| Mean CAL^*^, mm | 2.54 ± 0.89 | 2.63 ± 2.42 | 0.427 |
| Periodontitis^*^ |  |  | 0.051 |
| No, gingivitis | 36.8 | 40.9 |  |
| Stage I, II | 34.3 | 33.6 |  |
| Stage III | 22.2 | 13.6 |  |
| Stage IV | 6.7 | 11.8 |  |
| Tooth brushing ≤ 1 time^†^, % | 29.1 | 31.8 | 0.515 |
| No regular dental visit^‡^, % | 70.7 | 77.9 | 0.117 |
| Periodontal treatment^§^, % | 29.3 | 35.9 | 0.113 |
| Current smoking, % | 18.8 | 16.9 | 0.597 |
| Diabetes, % | 13.5 | 16.1 | 0.430 |
| Obesity (BMI ≥ 25.0), % | 25.6 | 23.5 | 0.553 |
| Hypertension definition (American), % | 58.2 | 62.5 | 0.329 |
| Serum triglycerides, mg/dL | 124.4 ± 100.9  100.0 (72.0, 141.0) | 123.0 ± 88.7  104.5 (68.0, 149.0) | 0.757 |
| Serum HDL cholesterol, mg/dL | 67.4 ± 17.5 | 68.3 (21.1) | 0.652 |
| Occupational status |  |  | 0.098 |
| Clerical support workers, % | 27.9 | 19.4 |  |
| Other jobs, % | 24.0 | 25.4 |  |
| Homemaker, unemployed or retired, % | 48.1 | 55.2 |  |
| All variables except serum triglycerides are given as the mean ± standard deviations or as a percentage. Serum triglycerides are given as mean ± standard deviations and median (first quartile, third quartile). | | | |
| Chi-square test was performed for categorical variables, and t-test was performed for continuous variable except serum triglycerides. Mann-Whitney *U* test was performed for serum triglycerides. | | | |
| ^*^ Excluding edentulous (n = 21 in participants who had missing data) | | | |
| ^†^ Excluding individual with missing value (n = 7 in participants who had missing data) | | | |
| ^‡^ Excluding individual with missing value (n = 32 in participants who had missing data)  ^§^ Excluding individual with missing value (n = 5 in participants who had missing data) | | | |
| DFT, decayed and filled teeth; PPD, probing pocket depth; CAL, clinical attachment level; BMI, body mass index; HDL, high-density lipoprotein. | | | |
