# Supplementary material for: Baseline periodontal status and modifiable risk factors are associated with tooth loss over a 10‐year period: Estimates of population attributable risk in a Japanese community
Source: J Periodontol. 2022 Feb 3;93(4):526–36. doi: 10.1002/JPER.21-0191 (PMC9305417; doi:10.1002/JPER.21-0191)
Supplement: Supplementary file 2 — Supplementary material [file JPER-93-526-s006.docx]

| Supplementary Table 2. Validation parameters for tooth loss | | | |  |  |
| --- | --- | --- | --- | --- | --- |
| Model | Sensitivity (%) | Specificity (%) | PPV (%) | NPV (%) | Area under the curve |
| All^*^ | 84.4 | 66.3 | 34.8 | 95.2 | 0.841 |
| Men^†^ | 78.2 | 70.6 | 41.6 | 92.3 | 0.822 |
| Women^†^ | 87.1 | 71.5 | 34.7 | 97.0 | 0.848 |
| ^*^ Model included age, sex, occupational status, diabetes, number of present teeth, periodontitis, number of DFT, toothbrushing frequency, no regular dental visit, periodontal treatment, current smoking, and obesity. | | | | | |
| ^†^Model included age, occupational status, diabetes, number of present teeth, periodontitis, number of DFT, toothbrushing frequency, no regular dental visit, periodontal treatment, current smoking, and obesity. | | | | | |
| PPV, positive predictive value; NPV, negative predictive value; DFT, decayed and filled teeth. | | | | | |
