# Supplementary material for: Baseline periodontal status and modifiable risk factors are associated with tooth loss over a 10‐year period: Estimates of population attributable risk in a Japanese community
Source: J Periodontol. 2022 Feb 3;93(4):526–36. doi: 10.1002/JPER.21-0191 (PMC9305417; doi:10.1002/JPER.21-0191)
Supplement: Supplementary file 3 — Supplementary material [file JPER-93-526-s008.docx]

| Supplementary Table 3. Descriptive statistics in men and women | | | |
| --- | --- | --- | --- |
| Baseline variables | Men  (n = 630) | Women  (n = 836) | p value |
| Tooth loss during 10 years, % | 21.1 | 14.8 | 0.002 |
| Age, years | 58.7 ± 9.6 | 57.9 ± 9.5 | 0.117 |
| Number of present teeth excluding third molar | 24.3 ± 4.6 | 24.3 ± 4.5 | 0.934 |
| Number of decayed and filled teeth | 13.1 ± 5.9 | 15.5 ± 4.9 | < 0.001 |
| Mean PPD, mm | 2.42 ± 0.80 | 2.10 ± 0.61 | < 0.001 |
| Mean CAL, mm | 2.81 ± 1.00 | 2.34 ± 0.74 | < 0.001 |
| Periodontitis |  |  | < 0.001 |
| No | 30.3 | 41.8 |  |
| Stage I, II | 30.6 | 37.1 |  |
| Stage III | 29.8 | 16.4 |  |
| Stage IV | 9.2 | 4.8 |  |
| Tooth brushing ≤ 1 time, % | 42.9 | 18.7 | < 0.001 |
| No regular dental visit, % | 72.2 | 69.5 | 0.257 |
| Periodontal treatment, % | 31.3 | 27.8 | 0.143 |
| Current smoking, % | 34.1 | 7.2 | < 0.001 |
| Diabetes, % | 16.7 | 10.9 | < 0.001 |
| Obesity (BMI ≥ 25.0), % | 29.8 | 22.9 | 0.003 |
| Occupational status |  |  | < 0.001 |
| Clerical support workers, % | 42.4 | 17.0 |  |
| Other jobs, % | 34.8 | 15.9 |  |
| Homemaker, unemployed or retired, % | 22.9 | 67.1 |  |
| All variables are given as the mean ± standard deviations or as a percentage. | | | |
| Chi-square test was performed for categorical variables, and t-test was performed for continuous variable. | | | |
| PPD, probing pocket depth; CAL, clinical attachment level; BMI, body mass index. | | | |
