# Supplementary material for: Baseline periodontal status and modifiable risk factors are associated with tooth loss over a 10‐year period: Estimates of population attributable risk in a Japanese community
Source: J Periodontol. 2022 Feb 3;93(4):526–36. doi: 10.1002/JPER.21-0191 (PMC9305417; doi:10.1002/JPER.21-0191)
Supplement: Supplementary file 4 — Supplementary material [file JPER-93-526-s003.docx]

| Supplementary Table 4. Number of present teeth lost according to periodontitis stage. | | |
| --- | --- | --- |
|  | Number of teeth lost | |
| Periodontitis stage | Mean ± SD | Median (first quartile, third quartile) |
| Periodontitis |  |  |
| No, gingivitis | 0.76±1.45 | 0 (0, 1) |
| Stage I, II | 1.65±2.23 | 1 (0, 2) |
| Stage III | 2.70±3.07 | 2 (0, 4) |
| Stage IV | 5.52±3.85 | 5 (3, 8) |
