# Supplementary material for: Baseline periodontal status and modifiable risk factors are associated with tooth loss over a 10‐year period: Estimates of population attributable risk in a Japanese community
Source: J Periodontol. 2022 Feb 3;93(4):526–36. doi: 10.1002/JPER.21-0191 (PMC9305417; doi:10.1002/JPER.21-0191)
Supplement: Supplementary file 5 — Supplementary material [file JPER-93-526-s001.docx]

| Supplementary Table 5. Regression coefficients obtained from the zero-inflated Poisson regression model | | | | | | |
| --- | --- | --- | --- | --- | --- | --- |
|  | All | | Men | | Women | |
| Baseline risk factors | β (SE) | p-value | β (SE) | p-value | β (SE) | p-value |
| Periodontitis |  |  |  |  |  |  |
| Stage III | 0.51 (0.05) | <0.001 | 0.46 (0.07) | <0.001 | 0.57 (0.07) | <0.001 |
| Stage IV | 0.47 (0.05) | <0.001 | 0.71 (0.11) | <0.001 | 0.34 (0.10) | <0.001 |
| Number of DFT | 0.04 (0.01) | <0.001 | 0.03 (0.01) | <0.001 | 0.06 (0.01) | <0.001 |
| ≤1 time tooth brushing | -0.07 (0.04) | 0.126 | -0.04 (0.06) | 0.527 | -0.15 (0.08) | 0.061 |
| No regular dental visit | 0.12 (0.05) | 0.016 | 0.08 (0.07) | 0.262 | 0.12 (0.07) | 0.079 |
| Periodontal treatment | 0.25 (0.04) | <0.001 | 0.25 (0.06) | <0.001 | 0.25 (0.06) | <0.001 |
| Current smoking | 0.23 (0.06) | <0.001 | 0.26 (0.07) | 0.001 | 0.11 (0.13) | 0.391 |
| Obesity (BMI≥25.0) | 0.13 (0.05) | 0.004 | -0.05 (0.07) | 0.461 | 0.32 (0.07) | <0.001 |
| Zero-inflated Poisson regression models; number of teeth lost as a continuous count variable was the dependent variable and risk factors were the independent variable. | | | | | | |
| All models included age, sex, occupational status, number of present teeth, diabetes, and all risk factors. | | | | | | |
| DFT, decayed and filled teeth; BMI, body mass index; SE, standard error. | | | | | | |
