# Supplementary material for: Baseline periodontal status and modifiable risk factors are associated with tooth loss over a 10‐year period: Estimates of population attributable risk in a Japanese community
Source: J Periodontol. 2022 Feb 3;93(4):526–36. doi: 10.1002/JPER.21-0191 (PMC9305417; doi:10.1002/JPER.21-0191)
Supplement: Supplementary file 6 — Supplementary material [file JPER-93-526-s002.docx]

| Supplementary Table 6. Oral condition and health behavior in 2007 and 2017. | | | |
| --- | --- | --- | --- |
| Variables | 2007 | 2017 | p value^*^ |
| Periodontitis^†^ |  |  | <0.001 |
| No, gingivitis, stage I, and II | 71.1 | 80.9 |  |
| Stage III | 22.2 | 12.4 |  |
| Stage IV | 6.7 | 6.6 |  |
| Number of DFT | 14.4 ± 5.5 | 14.0 ± 5.6 | 0.031 |
| No regular dental visit^‡^ | 70.7 | 47.9 | <0.001 |
| Tooth brushing ≤ 1 time^§^ | 29.1 | 22.2 | <0.001 |
| All variables are given as a percentage or the means ± standard deviations. | | | |
| ^*^ Compared between the survey in 2007 and in 2017. | |  |  |
| ^†^ Excluding individual with missing value (n = 8 in 2017) | |  |  |
| ^‡^ Excluding individual with missing value (n = 56 in 2017) | |  |  |
| ^§^ Excluding individual with missing value (n = 21 in 2017) | | | |
