# Supplementary material for: Baseline periodontal status and modifiable risk factors are associated with tooth loss over a 10‐year period: Estimates of population attributable risk in a Japanese community
Source: J Periodontol. 2022 Feb 3;93(4):526–36. doi: 10.1002/JPER.21-0191 (PMC9305417; doi:10.1002/JPER.21-0191)
Supplement: Supplementary file 7 — Supplementary material [file JPER-93-526-s004.docx]

| Supplementary Table 7. Association of changes in risk factors and tooth loss | | |
| --- | --- | --- |
|  | Adjusted OR (95% CI) | |
| Time-varying variables | Model 1 | Model 2 |
| Periodontitis (ref: no, gingivitis, stage I, and II) |  |  |
| Stage III | 0.75 (0.53-1.07) | 0.97 (0.69-1.39) |
| Stage IV | 9.67 (6.92-13.52) | 6.24 (4.44-8.77) |
| No regular dental visit (ref: yes) | 0.92 (0.75-1.13) | 0.82 (0.65-1.02) |
| Tooth brushing ≤ 1 time (ref: ≥2 times) | 1.07 (0.84-1.36) | 1.08 (0.83-1.41) |
| Logistic mixed models; tooth loss was the dependent variable and changes in risk factors were the independent variable. | | |
| Model 1 included age, sex, and one time-varying factor. | |  |
| Model 2 included age, sex, number of present teeth at baseline, and all time-varying variables such as periodontitis, no regular dental visit, and tooth brushing frequency. | | |
| OR, odds ratio; CI, confidence interval; ref, reference; DFT, decayed and filled teeth. | |  |
