# Supplementary material for: Baseline periodontal status and modifiable risk factors are associated with tooth loss over a 10‐year period: Estimates of population attributable risk in a Japanese community
Source: J Periodontol. 2022 Feb 3;93(4):526–36. doi: 10.1002/JPER.21-0191 (PMC9305417; doi:10.1002/JPER.21-0191)
Supplement: Supplementary file 8 — Supplementary material [file JPER-93-526-s009.pdf]

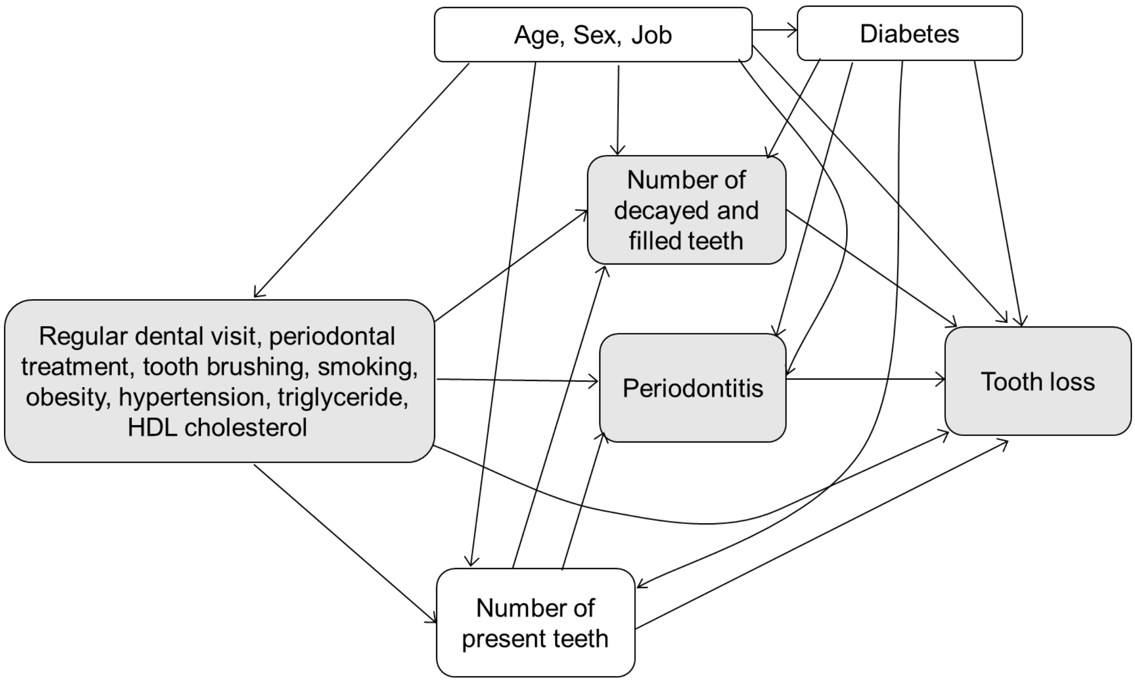

Supplementary Figure 1. Directed acyclic graph (DAG) representing presumed the relationship of periodontitis, tooth loss, and related-factors.

DAG was used to identify the minimally sufficient adjustment sets of variables. Nodes represent variables and arrows represent causal associations. Gray-colored nodes represent the causal effect of interest, which is tooth loss as outcome, periodontitis, number of decayed and filled teeth, regular dental visit, periodontal treatment, tooth brushing, smoking, obesity, hypertension, triglyceride, and HDL cholesterol as exposure.
