# Supplementary figures and images for: Baseline periodontal status and modifiable risk factors are associated with tooth loss over a 10‐year period: Estimates of population attributable risk in a Japanese community
Source: J Periodontol. 2022 Feb 3;93(4):526–36. doi: 10.1002/JPER.21-0191 (PMC9305417; doi:10.1002/JPER.21-0191)

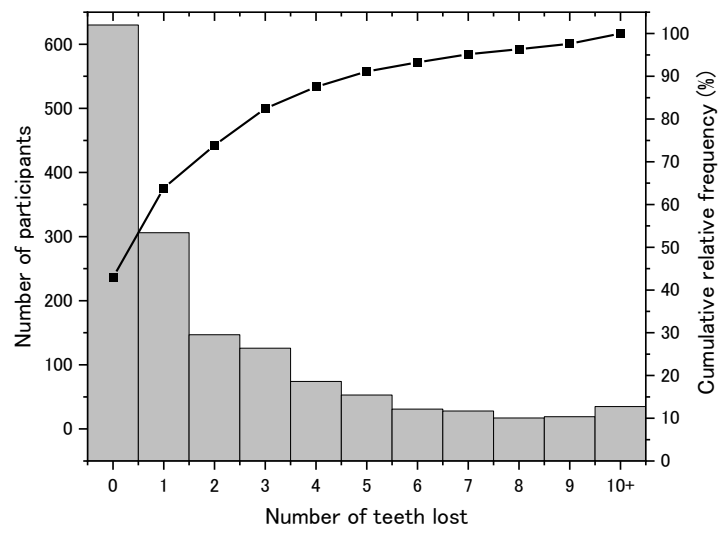

Supplementary Figure 2. Number of teeth lost during 10 years.

Supplement: Supplementary file 9 — Supplementary material [file JPER-93-526-s007.pdf]
